# Exploring the uptake and framing of research evidence on universal screening for intimate partner violence against women: a knowledge translation case study

**DOI:** 10.1186/1478-4505-11-13

**Published:** 2013-04-12

**Authors:** C Nadine Wathen, Jennifer CD MacGregor, Shannon L Sibbald, Harriet L MacMillan

**Affiliations:** 1 Faculty of Information and Media Studies, The University of Western Ontario, 1151 Richmond St., London, ON, N6A 5B7, Canada; 2 Faculty of Health Sciences, The University of Western Ontario, 1151 Richmond St., London, ON, N6A 5B7, Canada; 3 Departments of Psychiatry and Behavioural Neurosciences, and of Pediatrics, Chedoke Site, CH-P2, McMaster University, L8N 3Z5, Hamilton, ON, Canada

**Keywords:** Citation analysis, Domestic violence, Knowledge translation, Practice guidelines, Research utilization

## Abstract

**Background:**

Significant emphasis is currently placed on the need to enhance health care decision-making with research-derived evidence. While much has been written on specific strategies to enable these “knowledge-to-action” processes, there is less empirical evidence regarding what happens when knowledge translation (KT) processes do not proceed as planned. The present paper provides a KT case study using the area of health care screening for intimate partner violence (IPV).

**Methods:**

A modified citation analysis method was used, beginning with a comprehensive search (August 2009 to October 2012) to capture scholarly and grey literature, and news reports citing a specific randomized controlled trial published in a major medical journal on the effectiveness of screening women, in health care settings, for exposure to IPV. Results of the searches were extracted, coded and analysed using a multi-step mixed qualitative and quantitative content analysis process.

**Results:**

The trial was cited in 147 citations from 112 different sources in journal articles, commentaries, books, and government and news reports. The trial also formed part of the evidence base for several national-level practice guidelines and policy statements. The most common interpretations of the trial were “no benefit of screening”, “no harms of screening”, or both. Variation existed in how these findings were represented, ranging from summaries of the findings, to privileging one outcome over others, and to critical qualifications, especially with regard to methodological rigour of the trial. Of note, interpretations were not always internally consistent, with the same evidence used in sometimes contradictory ways within the same source.

**Conclusions:**

Our findings provide empirical data on the malleability of “evidence” in knowledge translation processes, and its potential for multiple, often unanticipated, uses. They have implications for understanding how research evidence is used and interpreted in policy and practice, particularly in contested knowledge areas.

## Background

It has been over 20 years since intimate partner violence (IPV) was declared to be a major public health problem [1], and while data on the prevalence [2-5], consequences [6-10], and costs [11-14] are well-established, many gaps remain in the knowledge base regarding how the health care sector can best detect and respond to IPV. These gaps have led to debates in the field and conflicting advice to health and social service providers, and policy decision-makers [15,16].

One of the most contested areas is whether or not all women should be routinely screened by a health care provider for exposure to IPV. Proponents argue that IPV’s burden of suffering necessitates universal screening of all women presenting to healthcare settings [17,18] with the hope that this will lead women on a path of help-seeking, with eventual reductions in violence and its health consequences. A number of evidence-based reviews and guidelines have concluded that the lack of evidence regarding the benefits, and potential harms and costs, of such screening on women’s health and well-being favours a case-finding approach (assessment of a patient based on risks for, and/or clinical signs or symptoms of, exposure) [19-21]. A recent updated systematic review for the US Preventive Services Task Force concluded that, while there may not be evidence from screening trials indicating benefit to women, the fact that screening can identify women, and that some intervention studies show promise for some women, warrants inclusion of universal screening protocols in health care settings [22].

This situation is typical of many health topics, where there is imperfect yet evolving research evidence, and a variety of interested and invested stakeholders who develop, promote and/or enact specific policies or practices, and who may or may not wish these options to be informed by “evidence” [23]. What makes this case interesting for empirical analysis is the increased policy activity in the field in 2011–12, especially in, but not limited to, the USA.

### The “case” – a randomized controlled trial of IPV screening

In 2009, in the *Journal of the American Medical Association (JAMA),* the results of a multi-site Canadian randomized controlled trial (RCT) were published indicating that universal screening for IPV did not significantly reduce women’s exposure to violence, or improve health outcomes or quality of life [24] (hereafter referred to as ‘the IPV screening trial’ or ‘the trial’). This was accompanied by an editorial recommending that until screening is shown to have measurable benefits for abused women, a case-finding approach, as defined above, is the best clinical response [25]. The key messages arising from the trial are outlined below. During the course of the current analysis, a second large RCT, conducted in the USA and also addressing IPV screening in health care settings, was published in *JAMA*, with very similar findings [26]; it too had an accompanying editorial re-emphasizing the need for clinical case-finding [27].

Given the debate surrounding this issue, we sought to examine how the evidence from the initial trial, published in a widely-read medical journal, has become represented in the literature, and to what extent it has influenced practice guidelines and policies. The trial – the largest of its kind at the time providing direct evidence regarding the effectiveness of screening in health care settings – also came at a significant moment in the evolution of the debate regarding the health care response to IPV, especially in the USA, where, as we will describe, recent clinical and legislative bodies have taken a position on this issue.

### Summary of key messages arising from the IPV screening trial

Key findings:

•All women in the trial showed reductions in exposure to violence across time – these were not associated with screening.

•Small differences between groups on life quality and depression were not statistically significant when the analysis accounted for women lost to follow-up, nor were they clinically meaningful. There were no differences in women’s health outcomes.

•Screened and control group women had no differences in the frequency of using violence-related health and social services.

•Screening may over-identify women as experiencing IPV, and many women must be screened to identify one woman who discloses abuse.

•There were no short-term direct harms of screening as implemented in this RCT.

•Sample attrition was a concern, and data analysis that accounted for these losses to follow-up further reduced differences between groups.

Trial conclusions:

•Health care providers and settings should be alert to the signs and symptoms associated with IPV exposure and ask questions about abuse when these are present (clinical case finding), ensuring that women are asked in sensitive ways to help identify their needs and safety concerns. Further, health care settings should develop and implement protocols for referral of abused women, according to their needs, to local services.

### Framing the case study: knowledge translation

Significant emphasis is currently placed on the need to enhance health decision-making processes with the best research-derived evidence available regarding specific health care practices and/or policies [28]. Indeed, most research funding agencies now require explicit “knowledge translation” (KT) (or “dissemination and implementation”) plans, including how “knowledge users” might be involved in research, from inception to communication. Numerous potential strategies exist to bridge the “know-do” gap and this area has grown into its own field of study [29-32]. However, a number of authors have started to question some of the assumptions that underlie the rationale of KT, including basic concepts, such as the nature of the “evidence” or knowledge that is supposed to be “translated” [33], as well as the role of context, and user values and beliefs, in influencing behaviours and decisions [34,35].

Thus, while much has been written on specific strategies to enable “knowledge-to-action” processes, there is less empirical evidence regarding what happens when these processes do not follow the proscribed logic, or yield unanticipated outcomes. One concept we explore is whether evidence can be thought of as “malleable”, that is, open to shaping and changing in ways that may or may not fit with its initial presentation. The present paper provides an empirical KT case study using the area of health care screening for IPV. Our specific research questions were: 1) how have authors and organizations interpreted and used the trial report? and, 2) how has it influenced practice guidelines and policies?

## Methods

No single validated method exists for conducting the kind of citation identification and analysis process we deemed necessary for understanding the broad representation and use of the IPV screening trial results; assessing research use in various contexts is a notoriously difficult task [36,37]. Therefore, we created a comprehensive search and analysis strategy, which we call a ‘modified citation analysis’, to capture both scholarly and grey literature, including news reports, using aspects of the method described by Jones et al. [36]. Traditional citation analysis is a widely used bibliometric tool used to examine links, within the scholarly literature, among published works [38]. While there are flaws to this method of analysis (*e.g.*, undervaluing recent papers) [39,40], it has been the basis for platforms such as Web of Science and Google Scholar.

Our modified approach included three main steps to collect citations from: 1) scholarly/academic peer-reviewed sources; 2) grey literature, defined as “that which is produced on all levels of governmental, academic, business and industry in print and electronic formats, but which is not controlled by commercial publishers” [41], p. 2, which is often difficult to find through conventional search tools; and 3) news reports. Keywords varied depending on the tool, website, or database used and included: “MacMillan”, “*JAMA*” and/or “*Journal of the American Medical Association*”, “violence”, “screening”, “2009”. The only inclusion criterion was that the source needed to cite the IPV screening trial. Dissertations, although considered grey literature, were excluded, as they were, in themselves, unlikely to be influential in terms of practice or policy considerations, especially in the context of the data set, which was almost exclusively composed of studies conducted in high-income countries. Conference proceedings were also excluded due to the difficulty of searching and obtaining presentation documents that may have included the relevant citation. Although we included books that came up in our searches and searched several full-text online book databases (*e.g.*, Google Books) we did not search for this particular source format exhaustively as no single database allows a comprehensive search of the references of all published books.

**Figure 1:**
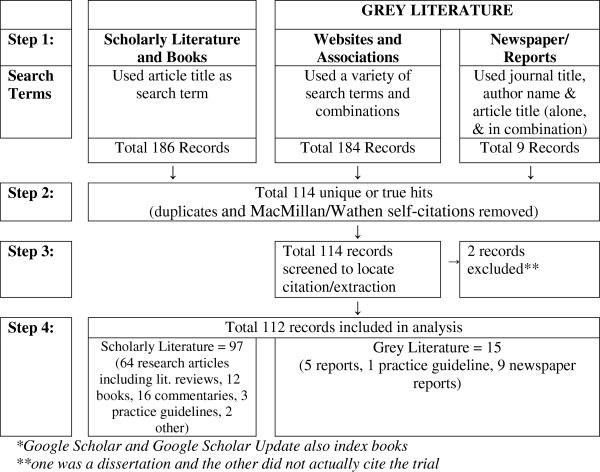
Search results flow diagram.

Searches included works published between August 2009 (when the trial was published) and October 2012. In Step 1, we began searching the scholarly literature by using Web of Science [42], a widely recognised citation searching tool accessing thousands of journals across multiple scholarly disciplines, including those relevant to this topic. We also used *JAMA*’s ‘cited by’ tool which allowed us to easily search for *JAMA* and related American Medical Association (AMA) Archives journal articles citing the trial, as well as Google Scholar (which has a ‘cited by’ tool), Google Scholar updates (which automatically emailed us relevant journal articles or books), and Scopus. In Step 2, we searched the grey literature using a targeted search of a variety of inter- and cross-disciplinary database search engines that feature both academic and grey literature (including MedLine Plus, MDConsult, UpToDate, etc.). A general Google search was also conducted (not reported) to ensure nothing was missed (see Additional file 1 for a complete list of databases searched and search results, including all cited sources). We also ‘hand searched’ the websites of those major healthcare professional associations (*e.g.*, AMA) and organizations (*e.g.*, American Academy of Family Physicians) that were likely to include IPV-related content. In Step 3, we searched news reports, using key news databases including Factiva, Lexis Nexis, Google News, and Proquest Canadian Major Dailies (see Figure 1 for search flow diagram).

All relevant sources were stored in the online reference management program RefWorks. Coding was conducted in three phases, to meet separate, but related, goals of developing a broad understanding of how the IPV screening trial was represented both explicitly and implicitly, and to interpret this in the broader context of the IPV evidence base and emerging practice and policy guidance. In Phase I, we wanted to explore how the IPV screening trial was used explicitly in each source. To do this we copied, verbatim, into a spreadsheet the text from each source (ranging from phrases to multiple paragraphs) that explicitly cited the trial; where a source cited the trial more than once, each section of text was extracted separately for analysis (that is, one source could have multiple extractions). Phase I analysis of the text took place in three steps. First, using content analysis [43], two team members (SS and JM; neither of whom were associated with the original IPV screening trial) independently coded all extractions to create a coding scheme. The coding scheme was created inductively, and guided by the extractions. Once the coding scheme was created, all extractions were re-coded (by JM and SS). The coding scheme had nine thematic codes (such as harm/benefit and methodology), with 26 sub-codes (such as no harm, no benefit and screening debate). Each extraction was coded to reflect how the trial was being used (for example to support screening or to provide evidence for no harm of screening). Any extraction that cited the trial’s major finding of ‘no benefit’, and/or of ‘no harm’, was also coded to include further explanation (if any) regarding the implications of this finding. All coding was consolidated between the two coders; disagreements between coders were resolved through re-review and discussion of the extracts. Potential quotes/extractions that could be used in the qualitative analysis to emphasise these themes were pre-selected (by JM and SS) from the data set without involvement of NW or HM. Final decisions on which of these was included in the manuscript were made by the lead author (NW).

In Phase II, we looked at the source itself (*i.e.*, where the extraction came from) and coded each source in two steps. First, each source (based on the title, abstract, or full-text when no abstract was available) was coded as either mainly focused on IPV screening or not. Next, relevant text (*i.e.*, on the topic of IPV screening, often from the Conclusions or Summary) was extracted from each source. Using a directed coding approach, each source was categorized into one of four categories: supports screening, does not support screening, unclear, or no position. Where appropriate, sources were then further inductively coded into explicit support or non-support, implicit support or non-support. To do this, the content, including the research question and/or use of language, was examined and interpreted by the two coders to either imply support or not, or other more specific categories (such as: implied based on use in one specific target population). In the case of coding disagreements (which were rare), both coders went back to the original source or extraction to re-code material that caused the disagreement, and a final code was decided by consensus.

Finally, in Phase III, and consistent with the variability in how “screening” is defined and discussed, we examined how each source used the term “screening”, and inductively coded whether or not the term was explicitly defined. Only sources that had an explicit definition of screening were further coded (*e.g.*, screening defined as healthcare professional routinely asking all women about abuse).

For all three phases of coded results, descriptive statistics (frequencies) were generated using SPSS 20.0. Details of the coding process, the search results, and the full bibliographic details of all sources citing the IPV screening trial are found in Additional file 1.

## Results

We present the results in two ways. First, data are quantitatively summarized in Tables 1 and 2 to address the research question regarding the number and types of sources that cite the trial, and specific ways that this was done in the context of those sources. Next, themes are presented that emerged from the analysis regarding how the IPV screening trial was represented; we used extracted text from the sources to exemplify and begin to interpret these themes more qualitatively.

**Table 1:** Summary of characteristics of citing sources

**Source type**	**n (% of 112 sources)**
Research article	56 (50%)
Commentary	16 (14.3%)
Books or book chapters	12 (10.7%)
News reports	9 (8%)
Practice guidelines	4 (3.6%)
Grey literature (*e.g.*, government) reports	5 (4.5%)
Literature review (non-systematic)	4 (3.6%)
Literature review (systematic)	4 (3.6%)
Other	2 (1.8%)
**Source content regarding IPV screening**	**n (%) (of 112)**
IPV Screening focus (yes)	55 (49.1%)
Define screening (yes)	13 (11.6%)
Support universal screening	35 (31.3%)
Do not support universal screening	28 (25%)
Unable to determine/no position on screening	49 (43.8%)
**Times citing trial (within source) (range 1–5)**	**n (%) (of 112)**
Once	90 (80.4%)
2-3 times	21 (18.8%)
5 times	1 (0.9%)
**Citation location (within source)**	**n (% of 147 total extractions)**
Discussion	49 (33.3%)
Introduction/Background	35 (23.8%)
Method	4 (2.7%)
Results	4 (2.7%)
Unable to specify (non-sectioned source)	55 (37.4%)

**Table 2:** How the IPV screening trial was cited (147 extractions from 112 sources)

**Code/Sub-code**	**# sources given code (at least once) (% of 112)**	**# times code used for extraction (% of 147)**
**1. Major finding (Harm/Benefit):** Used when IPV screening trial major finding cited:	61 (54.5)	80 (54.4)
1.1 No harm from screening	13 (11.6)	14 (9.5)
1.2 No benefit to screening	33 (29.5)	35 (23.8)
1.3 Both (no harm/no benefit)	20 (17.9)	20 (13.6)
1.4 Benefit to screening	8 (7.1)	8 (5.4)
1.5 Results inconclusive	2 (1.8)	2 (1.4)
**2. Methods/Measures/Statistics:** Used when IPV screening trial method or measure cited:	19 (17)	21 (14.3)
2.1 Women-centred outcomes	2 (1.8)	3 (2)
2.2 Multi-level modelling	1 (.9)	1 (.7)
2.3 Harms	3 (2.7)	3 (2)
2.4 CAS cut-off	2 (1.8)	2 (1.4)
2.5 WAST	2 (1.8)	3 (2)
2.6 Other methods/measures/statistics	9 (8)	9 (6.1)
**3. General screening/IPV reference:** Used to cite IPV screening trial for general point about screening or IPV:	43 (38.4)	51 (34.7)
3.1 Screening debate	15 (13.4)	16 (10.9)
3.2 Importance of IPV/screening discussion	2 (1.8)	2 (1.4)
3.3 HCP education/training	2 (1.8)	2 (1.4)
3.4 Other	6 (5.4)	7 (4.8)
3.5 Insufficient evidence to support screening	23 (20.5)	24 (16.3)
**4. Interventions/Services:** Used when IPV screening trial cited to show priority and/or research/knowledge gap in the area of interventions/services for IPV	13 (11.6)	14 (9.5)
**5. Minor findings:** Used when a minor IPV screening trial finding cited:	22 (19.6)	31(21.1)
5.1 Effect size	1 (.9)	1 (.7)
5.2 Retention rate	8 (7.1)	8 (5.4)
5.3 Women not talking to HCP	8 (7.1)	8 (5.4)
5.3a Women talking to HCP	4 (3.6)	5 (3.4)
5.4 Sensitivity	2 (1.8)	2 (1.4)
5.5 Other minor finding	7 (6.3)	7 (4.8)
**6. No specific citation:** Used when IPV screening trial listed as a reference or a source for readers to consult (but nothing specific cited in text)	3 (2.7)	3 (2)
**7. Incorrect:** Used when IPV screening trial cited incorrectly (*e.g.*, for a finding not actually reported)	2 (1.8)	3 (2)
**8. Other:** Used when no other code appropriate, especially when reason for citing IPV screening trial is unclear	2 (1.8)	2 (1.4)

Note: Each extraction received a maximum of three distinct codes. Some sources were given more than one sub-code per category. IPV: Intimate partner violence; HCP: Health care provider; CAS: Composite Abuse Scale [48]; WAST: Woman Abuse Screening Tool [49].

### How the IPV screening trial was cited, and in what context

In total, we found 147 instances of the trial being cited (“extractions”) in 112 different sources, as summarized in Table 1, Figure 1 and Additional file 1. In an effort to understand the context within which each citation was placed, we examined several aspects of each source with respect to its IPV-related content and its position on screening more specifically (Table 1). Almost half of the sources were focused on the issue of IPV screening (49%). Only 13 sources (12%) explicitly defined screening, 8 (15%) among those that focused on IPV screening, and 5 (9%) among those that did not. The definitions took many forms. For example, whereas some definitions included screening plus a follow-up intervention (*e.g.*, referral, brief counselling) as integral to the clinical screening process (n = 3, 23%), most did not (n = 10, 77%). Some, but not all, specified who should be asked (n = 6, 46%), for example, “women and adolescents” [44], p. 166, “asymptomatic persons,” [45], p. 725, or “all women or patients regardless of presumed risk” [48], p. 856. We also examined the position that each source took on IPV screening; 35 sources (31%) supported it, with support explicitly stated in 21 instances, six of which added a caveat to their support (*e.g.*, privacy of women must be ensured during a screening encounter). In the remaining 14 (13%) instances, support for IPV screening was implied through language or by the research methods, for example, when authors included the implementation of IPV screening as a positive outcome variable in their research [46]. Many sources were coded as not supporting universal IPV screening (25%), again, some explicitly so (4%), and others implying their position (21%), for example by explicitly supporting screening in a specific population only (*e.g.*, in reproductive or sexual health settings). In 10 instances (9%), coders were unable to determine the authors’ position (if any), and the remaining 39 (35%) were seen as taking no position, either because they did not address the issue of IPV screening, or because they presented no stance regarding screening.

Table 2 describes how these 147 individual extractions cited the trial. Over half (55%) of the sources cited the trial’s major findings of ‘no benefit of screening’, ‘no harm of screening’, or both, in at least one extraction (n = 35, 14, and 20 extractions, respectively). The trial was also cited for a variety of other reasons such as to support provider education or justify a statistical or methodological approach. A fairly common approach was to use the trial as a more general IPV or screening reference (35%). For example, some cited the trial for the general conclusion that there is insufficient evidence to recommend universal screening (16%) or for the fact that a debate regarding IPV screening exists (11%). Other extractions focused on secondary findings reported in the trial (21%), such as the number of women in the RCT who did or did not have an IPV discussion with their healthcare provider (9%) or the RCT’s retention rate (5%). In 10% of cases, the trial was cited to support the statement that there is a research priority or knowledge gap in the area of interventions/services for IPV.

Of the 14 citations referring to the trial’s finding of ‘no harms of screening,’ one further elaborated, stating the importance of considering whether ‘safety concerns’ as conceptualized by MacMillan et al. “adequately captures the possible harm associated with screening for IPV” [47], p. 7. The ‘no benefit of screening’ finding was further elaborated in 34% of instances (n = 12 of 35 citations). Authors commented on this finding by identifying methodological and/or statistical limitations in the trial; the following were all single instances of these comments, unless otherwise indicated: 1) that no designated intervention occurred after screening; 2) that the control group was also administered questions about IPV (*i.e.*, after the clinical visit; n = 2); 3) that the experiences (*e.g.*, of abuse or service access) of the women in the sample were too varied to find beneficial effects of screening; 4) poor screening method used; 5) the lack of designated intervention combined with the fact that most women accessed services prior to screening; 6) the lack of designated intervention combined with the fact that control women were also assessed, as part of the research process, on IPV exposure; or 7) attrition, sometimes combined with a comment about one of the following: statistical analysis methods, lack of a designated intervention, control group administered IPV questions or controlled conditions along with unspecified methodological problems.

Among the 20 citations referring to both ‘benefit’ and ‘harm’ findings, six were qualified by the authors. However, in four citations the qualification was specific to the ‘no benefit’ finding, not the ‘no harm’ finding (*i.e.*, highlighting the fact that no intervention was provided after screening, that the control group was exposed to the intervention (n = 2), or both). Of these four sources, three explicitly supported universal IPV screening and one supported screening in a specific population.

A key theme evident across most sources, especially those with an implicit or explicit pro-screening position, was to contest or offer an alternative explanation for the trial’s main finding of ‘no benefit’. As indicated above, this was most often done by referring to methodological limitations, sometimes through re-iteration of those articulated in the *JAMA* report [44,50], and other times through extrapolations of these limitations, as indicated in the following:And,

“… recent randomized trials suggest that screening does not reduce reabuse or lead to significant differences on other quality of life or safety outcomes (Koziol-McLain et al., 2010; MacMillan et al., 2009). On face-value such findings would suggest that there is little merit in screening; however high loss to follow up (MacMillan et al., 2009), and insufficient sample size for effect (Koziol-McLain et al., 2010) limit the robustness of these findings.” [51], p. 151.

“… methodological issues (*i.e.*, sample attrition and *exposure to the intervention in the control group*) [emphasis added] of a recent randomized, controlled trial rendered its findings inconclusive (MacMillan et al., 2009)…” [52], p. 6.

Notable in the second extraction is the statement that the control group was exposed to the intervention even though no control women were screened. These authors referred to the IPV screening trial findings as “inconclusive”, which was not the interpretation provided in the conclusion of the trial (nor in the accompanying editorial), but rather their own, a framing that occurred explicitly in at least one additional source [53], and was implied in several others.

Methodological and statistical concerns about the trial methods did not extend to the ‘no harms’ finding, however, and in a number of sources, authors endorsed this finding as support for screening. This is exemplified in the following extract:

“This study demonstrates a high level of endorsement among women for routine intimate partner violence screening and that no harm or adverse effects were linked with the intervention, which is consistent with recent work done by Houry et al. (83) and MacMillan et al. (65) Thus, the findings support the view that intimate partner violence screening intervention does not contravene the principle of nonmaleficence and, in the right circumstances, may be aligned with beneficence.” [54] p. 421.

Sometimes, there were contradictory interpretations of the utility of the trial findings within the same source:

“One of the few randomized studies available reported that victims whose positive screen results were communicated to their physicians had no better outcomes than women who were simply given a referral card (MacMillan et al., 2009). Unfortunately, the retention rate in this study was too low to support its general conclusion, no evidence was provided from either study on whether physicians actually used the information they got from the screen, and a debatable statistical method was used to neutralize the reduction in harms that were found. Interestingly, more than four times as many abused victims who were screened discussed violence with their physicians than abuse victims who were not screened (44% *versus* 10%), which demonstrates a remarkable effect of screening.” [55], p. 390.

Some sources seem to overlook certain aspects of the trial in favour of others when summarizing evidence, for example, the practice guidelines released by the Registered Nurses’ Association of Ontario referred to the IPV screening trial once in its recommendation supporting universal screening, as follows, with no mention of the lack of benefit finding:

“Furthermore, studies have shown that: no harm or adverse effects were linked with this type of questioning (Houry et al. 2004; Koziol-McLain et al., 2010; MacMillan et al., 2009).” [56], p. 3.

Some authors who cited the trial did not cite the major findings, but instead used the citation for different purposes. Of the 63 sources with a position on screening, 29% of those deemed supportive of screening and 46% of those deemed not supportive of screening did not cite either of the main findings specific to harm or benefit, and instead cited the trial for other reasons such as more minor findings (*e.g.*, retention rate) or the use of particular methods. In other cases, authors provided descriptions of the trial findings that seemed to contradict the actual results, while still supporting a pro-screening position:and,

“In a randomised trial MacMillan and colleagues recently confirmed that screening for domestic violence is safe and feasible and leads to significant improvements in quality of life, although the latter finding disappeared after adjustment for confounders.” [57], p. 408.

“Although a recently published randomized trial failed to demonstrate benefit from intimate partner violence screening, (25) most major medical societies continue to recommend routine screening of women for partner abuse. The need exists for further study of the effects of screening and the development of effective interventions. Until that time arrives, however, clinicians should continue to screen all their adult female patients for partner violence not only for compliance with national guidelines, but because its high prevalence and extensive health effects warrant routine inquiry.” [58], p. 1165.

A more nuanced approach was exemplified in the following extract, as well as in a small minority of other sources [59]:

“The MacMillan study (21) was important because it demonstrated the safety of IPV screening, but it is important to note that their screening intervention group did not receive any intervention beyond a referral, and the control group was also screened and referred only at the end of the visit as opposed to the beginning. This likely negated any ability to detect a difference but does not mean that screening and referral were ineffective since, unlike our results, IPV-identified women in both groups exhibited long-term reductions in IPV recurrence (21).” [60], p. 897.

Some authors interpreted the results in the broader context of an appropriate and meaningful response from health care settings and professionals to identify and, more importantly, respond to women experiencing violence. These types of uses of the evidence are exemplified by the following:And,

“Despite the lack of evidence supporting effectiveness of IPV screening (32, 33 (MacMillan et al., 2009)), there is compelling logic for screening (34,35), and routine assessment for victimization is supported by most major medical societies (36). However, requiring screening questions does not guarantee that clinicians consistently or effectively implement them, nor that they respond appropriately to disclosures of abuse (26). Our findings may reflect that mandating screening without providing effective and accessible means of intervening will have a limited impact on victims.” [61], p. 320.

“In summary, there continues to be a lack of evidence that universal screening alone improves health outcomes for IPV survivors. It is certainly understandable that clinicians and health care facilities have implemented universal screening programs, given the prevalence and potential severity of IPV. However, the results of the study by MacMillan et al. [6] should dispel any illusions that universal screening with passive referrals to community services is an adequate response to violence in intimate relationships. Specific interventions to prevent the recurrence of abuse for women at risk of violence should be implemented and rigorously tested, preferably in randomized trials, without further delay.” [25], p. 569.

## Discussion

The present study analysed the uptake and representation of a major new source of evidence in an important, yet complex and contested, health care area. In general, we found that even evidence from a large trial considered to be of “fair” quality (*i.e.*, having no major limitations) by the US Preventive Services Task Force [12] and published in a journal with wide circulation and an accompanying editorial clearly summarizing the findings, is not interpreted consistently in subsequent literature, including major clinical and public policy documents. In fact, the article was subject to a number of sometimes contradictory interpretations and uses, indicating the potential for evidence to be more malleable than might otherwise be expected. A number of strategies, both active and passive, were brought to bear to downplay the aspects of the trial findings (*i.e.*, no benefit of screening) when they were inconsistent with specific positions, *i.e.*, support for universal screening. These strategies included overstating or extending study limitations, while, in some cases at the same time, selectively appropriating and/or ignoring the same study limitations about findings more consistent with the stated position (*i.e.*, no harms of screening or that the screened group reported more discussion with a clinician). It was interesting that those who deemed the findings invalid by virtue of methodological flaws, and used this argument to refute the lack of benefit, could at the same time find the trial to have sufficient validity to support the finding of ‘no harms’ of screening.

We also examined sources citing the trial that provided specific clinical guidance regarding identification and/or referral of abused women in various clinical situations, or for different groups of women. Several of these were formal clinical practice guidelines (CPGs) prepared by established groups [50,56,62,63]; in some cases two or more documents related to the guideline (*i.e.*, evidence summaries, supplements, etc.) were published, and while all of these were included in the citation counts, it should be noted that the treatment of the IPV screening trial was the same in all documents. Other review articles [64,65] endeavoured to provide clinical guidance, but were not formal guidelines or recommendations endorsed by specific associations or other groups. Of note, some CPGs published after the trial did not cite it [17], relying instead on other guidelines [44] to support their positions.

Another finding that stands out is related to definitions and terminology; only 12% of the sources we analysed actually defined what they meant by screening. While the issue of providing a specific definition may seem pedantic, the implications of failing to clearly distinguish different ways to identify women exposed to violence likely underpins much of the confusion and debate in this area [27]. Consider the following notable example of how the lack of common definitions influences interpretation and use of evidence: the IPV screening trial was criticized because women in the control group were said by some to have been exposed to the intervention [44,51,52,60]. However, as reported in the trial, women in the control group were only asked questions about violence using self-completed research tools administered by a research assistant subsequent to their encounter with the clinician; this information was never passed on to the clinician. If, as some argue, asking any questions about violence, whether linked to a clinical encounter or not, is “screening”, then presumably simply answering any questions about health conditions, such as depression, diabetes, etc., linked or not to an encounter with a clinician, is considered screening. This way of thinking disregards the important element of the patient-clinician encounter in assessment and diagnosis, which would be expected to follow a positive screening result for any condition. In fact this issue was tested in the more recently published IPV screening trial [26], and no life quality or other differences were found between the unscreened group that was asked about violence and given violence resources, and the “true” control group that was followed but never exposed to violence questions or materials. At the other extreme are the several sources that defined screening to include not only having a health care provider ask about IPV, but also couple the screening with specific follow-up services.

Similarly, case-finding – assessment of a patient based on risks for, and/or clinical signs or symptoms of, exposure – is often called, in this literature, ‘targeted screening’ or ‘risk-based inquiry’. Since all professional associations and guidelines appear to agree that asking about abuse under these conditions is a minimum standard of care to better assess immediate safety, and also to help address potential co-morbid conditions, it is unclear why the concept of ‘screening’ needs to be invoked at all. We were struck by how many sources used the term screening when in fact they were discussing a different type of inquiry. For example, among the seven sources supporting inquiry in specific populations or settings where clients would likely be at higher risk for IPV, almost all used the term ‘screening’. Seeking clarity about these issues would be an important step in ensuring that policies and practices are consistent with principles of good clinical care, as well as being comparable across settings, both for clinical auditing purposes, and when conducting research and evaluation.

These debates about evidence are not simply esoteric. Recently, a report by the US Institute of Medicine (IOM) recommended universal screening, while acknowledging that the evidence supporting it remained limited [44], a finding reinforced in the 2012 evidence synthesis [22] and new recommendation (see below) on the topic by the US Preventive Services Task Force (USPSTF) [66]. The 2011 IOM Report was used as the basis for including IPV screening in the US Affordable Care Act [67], thereby embedding it in legislation.

However, it is interesting to note how the above prominent guideline documents situate the evidence, and specifically the IPV screening trial, with respect to the actual recommendations. Both the USPSTF and IOM guidelines are based on 1) the fact that screening instruments can identify abuse; 2) there is emerging evidence that specific interventions might work in some groups of women; and 3) that the IPV screening trial had methodological limitations. The IOM report stresses the issue of “exposure in the control group”, as described above. As they state, “women randomized to the unscreened comparison group were also asked questions about abuse, received information about intimate partner violence, and were offered services if needed, reducing measureable differences between screened and unscreened women” [44], p. 106. The USPSTF evidence review [22], raised similar issues, while providing a “fair” quality rating, which, applying their evidence rules, would normally indicate a recommendation in the opposite direction: fair evidence of no benefit would lead to a D recommendation, or, if concerns of generalizability were an issue (such as the fact that the IPV screening trial was done in Canada), perhaps an I statement of insufficient evidence [68]. However, the USPSTF recommendation is a B grade, stating that “clinicians screen women of childbearing age for intimate partner violence (IPV), such as domestic violence, and provide or refer women who screen positive to intervention services” [66]. While the IOM report, and the related ACA legislation, preceded the more recently published RCT of IPV screening in USA health settings that also found no benefit of screening [26], the USPSTF guideline was published approximately 5 months after publication of this new trial [66].

From a strictly ‘evidence-based medicine’ perspective, these decisions may be seen as unusual, or at best a modification of the usual ‘rules of evidence’ [68] to allow certain forms of evidence to be more influential than they normally would. However, from the perspective of the policy studies literature specific to research utilization, and an emerging and more nuanced examination of ‘knowledge translation’ and ‘implementation science’ practices [35], these processes and outcomes become less confusing (though no more ‘evidence-based’). Building on Weiss’ notion of tactical use of research [69], Greenhalgh and Wieringa [23], p. 507 eloquently state: “…research evidence may be used instrumentally and rhetorically to back-up particular value-based positions. This occurs particularly when there is ‘high issue polarisation’ – that is, disagreement among stakeholders about what the significant problems are and how they might be addressed.” The notion of ‘evidence-in-context’ is emerging, regardless of topic area, as the only way to truly understand the ‘translation’ of ‘knowledge’ to policy and practice; trying to assess the impact of ‘evidence’ without a concurrent evaluation of the context(s) in which it is to be used increases the likelihood of lack of success of knowledge translation strategies [34,70,71].

In the context of our case example, decision-makers, researchers, and clinicians are actively engaged in determining how best to identify and help abused women when they present to health care settings. The notion of screening was introduced in the early 1990s to address what the AMA at that time termed “a major public health problem” [1], and screening was seen as a way to sensitize clinicians to the issue, have them identify women, and start a process of care and referral. However, as indicated above, the concept of screening is understood in different ways, and the reality of adding this clinical activity to the broad range of actions that primary care clinicians (whether in the emergency department or a family practice) are already required to do, with little guidance on what happens after identification (and little formal training in the issue), is where the “issue polarisation” described by Greenhalgh and Wieringa [23] can be seen to emerge.

At the same time, processes and technologies to routinize certain aspects of assessment and care became prominent, with a leading trend now being to have women complete questions about violence exposure (and other ‘lifestyle issues’) on computer kiosks in waiting areas [72,73]. Positioning the difficult task of responding to questions about abuse to a computer, in a room surrounded by other patients, seems counter to all principles of good care for women experiencing violence [74]. Given the resource implications for USA health care settings as they work to comply with ACA legislation and screen all women, it is conceivable that much of this screening will move to these computer-assisted models, with women who screen positive receiving print-outs of local resources. What happens to women after screening is not spelled out in the guidelines reviewed above. Additional evaluation of the implementation of this legislation in the USA is warranted.

### Limitations and future research

It is well acknowledged in the KT literature that one of the most difficult things to assess is the actual impact of new knowledge on specific policies or practices, or, ultimately, on health-related outcomes [34,75]. We have used a highly focused approach to assess the uptake and representation of a specific new research report in a range of documents, from news reports to national guidelines embedded in legislation. Our modified citation analysis was able to capture formal and informal published material in the time-span for the review; however, it would have missed documents, especially in the grey literature, unable to be located with our search processes. The analysis, and our interpretation, were limited in their ability to understand the ‘why’ and ‘how’ of the decisions that went into the use (or non-use) of the trial report – published reports rarely explicitly describe the decisions that underpin selection and use of evidence, whether by a sole author, or a consensus committee. Methods that can triangulate analysis of both the end products of knowledge use, as well as the decision processes, for example through qualitative and/or observational research with decision-makers as they interact with the new evidence and decide whether/how to use it, would enrich this kind of analysis.

It should also be noted that, in addition to the *JAMA* publication, the findings of the trial, and a series of related studies, were disseminated more broadly using a number of tailored and more general KT strategies. These are described in depth in other publications [76,77], and included knowledge-sharing events tailored to specific audiences (*i.e.*, policy briefings to Canadian government representatives, a clinical live chat via the *JAMA* Author-in-the-Room platform for a related study), general dissemination including press releases and media interviews, and a series of knowledge exchange forums with mixed groups of stakeholders in Canada (researchers, policy-makers, advocates, and clinical leaders in the area of family violence). The extent to which these other, generally more local, activities influenced use of the trial report in the documents described in this study are unknown, however they are likely minimal given the international scope of the literature included in this analysis.

It is important to highlight that two of the study authors were co-authors of the IPV screening trial, which formed the impetus for this analysis. To reduce potential bias in the data collection and analysis processes, these aspects of the study were conducted by two investigators not associated with the original IPV screening trial, who independently coded all extractions using established protocols for this type of content analysis, and who initially determined how these data were grouped into themes, including pre-selection of potential quotes that could be used in the qualitative analysis to emphasise (or provide contrary viewpoints) on these themes.

Finally, since articles on the topic not citing the trial would, according to the inclusion criteria, not be included, some might argue that we have under-reported the use of findings from the IPV trial. In other words, there are relevant articles that might well have cited this study in some capacity but chose not to. A search conducted to identify potentially high-impact documents such as guidelines and policy or position papers did yield some examples of this, including the practice guideline of the American Congress of Obstetricians and Gynecologists [17], and a “guidance memo” to all health professionals from the Director of the US Department of Health and Human Services’ National Health Resource Center on Domestic Violence and the Senior Public Policy Advocate of the advocacy group Futures Without Violence [18]. Both of these documents recommended screening, reinforcing the finding that some authors and organizations chose not to include the trial when formulating positions not consistent with its findings.

While the areas of health policy and clinical practice differ somewhat in terms of how they use evidence, as well as what kinds of evidence matter, much of the discourse in both areas assumes that these processes should include consideration of research and laments the lack of its uptake, especially when there are proven-effective interventions [78-80]. This paper highlights a different phenomenon – the push to develop public and clinical policy when the available evidence does not, on its face, support specific actions. While it is true that current thinking in the area of evidence-informed policy and practice promotes the use of higher levels of evidence (*i.e.*, systematic reviews of multiple trials, rather than individual studies) to support decisions [81], it does become a challenge to the ‘chain of evidence’ when these trials are mis- or un-represented in the evidence syntheses. More studies analysing this aspect of research mis- and non-utilization would assist in understanding whether our findings were specific to the context of IPV screening, or are seen more broadly. Similarly, while there is an emerging literature on the role of so-called ‘negative trials’ in health care – those that show no difference or negative outcomes related to the intervention [82] – and guidance is available regarding making policy decisions in the face of uncertain evidence [83], it is clear that it is difficult to engage decision-makers in using evidence [84]. Even when compelling evidence emerges demonstrating that an existing practice is questionable or even detrimental, established practices and policies may not change [82]. Indeed, many of the same features of evidence-informed decision-making processes highlighted in our analysis have been described in other policy-oriented KT studies [85]. The type of analysis conducted in this study might serve as a useful form of “reality-check” when assessing a new practice or policy – has the policy included consideration of all potentially relevant evidence, as well as the myriad other factors (including tacit knowledge, resource implications, contextual factors, etc.) that surround any such decision? The role of negative trials, and contrary evidence in general, in public and community health practice and policy, requires further research.

## Conclusions

Our findings highlight the importance of considering the malleability of research evidence and its potential for both intended, and unintended, uses. They provide empirical data regarding what many have written about regarding barriers to evidence-based decision-making and research utilization [23,34,69-71], they have implications for understanding how research evidence is taken-up and interpreted in policy and practice, and they can inform development of specific KT strategies in contested knowledge areas. Our analysis provides a good example of what Greenhalgh and Wieringa [23], p. 501, describe as “knowledge [that] obstinately refuses to be driven unproblematically into practice.”

## Abbreviations

AMA: American Medical Association; CPGs: Clinical practice guidelines; IOM: US Institute of Medicine; IPV: Intimate partner violence; JAMA: Journal of the American Medical Association; KT: Knowledge translation; RCT: Randomized controlled trial; USPSTF: US Preventive Services Task Force.

## Competing interests

No financial interests to declare. Two of the authors of the present study (CNW and HLM) were co-authors of the IPV screening trial that forms the basis of the analysis.

## Authors’ contributions

JCDM and SLS carried out the majority of data collection and analysis. CNW and HLM conceptualized and designed the study, and participated in data interpretation. All authors contributed to drafting and finalizing the manuscript, and approved the final version.

## Supplementary Material

Additional file 1 Methods Details: coding process, search results and source citations. Click here for file
