# Supplementary material for: Exploring the uptake and framing of research evidence on universal screening for intimate partner violence against women: a knowledge translation case study
Source: Health Res Policy Syst. 2013 Apr 12;11:13. doi: 10.1186/1478-4505-11-13 (PMC3637368; doi:10.1186/1478-4505-11-13)
Supplement: Additional file 1 — Methods Details: coding process, search results and source citations. [file 1478-4505-11-13-S1.doc]

**Additional File 1: Methods Details: Coding Process, Search Results and Source Citations**

1. Coding Process

| **PHASE** | **STEPS** | **Details** | **Outcome** |
| --- | --- | --- | --- |
| I | 1. Coding Framework Development (inductive) | All extractions read independently by 2 researchers | Coding Framework with 9 thematic codes and 26 sub-codes |
|  | 1. a) All extractions coded using coding framework | All extractions read and coded independently by 2 researchers |  |
|  | b) All extractions referencing ‘no harm’ or ‘no benefit’ finding were further coded. | When extractions were coded as having cited the trial for the ‘no harm’ or ‘no benefit’ finding we further coded to see if explained by the authors |  |
|  | 1. Consolidated coding | Researchers worked together to create one set of coded work. Disagreements resolved through discussion. | One set of coded data to be inputted in SPSS for frequency analysis |
| II | 1. Coded source as either focused or not on IPV screening | Each source was examined on the whole to see if the focus of the source was IPV screening | Sources that did have a focus on IPV screening were counted |
|  | 1. Coded sources support or not of screening | Each source was categorized into one of four categories: supports screening, does not support screening, unclear, or no stance |  |
| III | 1. Explore explicit definitions of screening | Each source coded as containing explicit definition or not; explicit definitions extracted. | A list of explicit definitions for screening |
|  | 1. Implicit coding of screening definitions | Each definition was read and coded inductively (coding list was created based on data) | Groups of similar definitions |

**2: Search Results Details**

NOTE: due to overlap across sources, decisions were made to assign a given source to a specific category for the data presented in Figure 1 (main text). Therefore, the values below, and those in Figure 1, may not be exactly the same.

**Scholarly Literature Sources**

| **Name of Database/Search Engine** | **Hits** | **Unique Hits** |
| --- | --- | --- |
| Web of Science | 65 | 34 |
| Google Scholar | 106 | 35 |
| *Journal of the American Medical Association*’s ‘cited by’ tool | 4 | 0 |
| Google Scholar Update | 11 | 11 |
| **TOTAL HITS** | **186** | **80** |

**Scholarly + Grey Literature Sources**

| **Name of Database/Search Engine** | **Hits** | **Unique Hits** |
| --- | --- | --- |
| Scopus (grey and scholarly) | 75 | 7 |
| Medline plus | 0 | - |
| Science direct | 8 | 0 |
| Google Books | 6 | 6 |
| MDConsult | 3 | 1 |
| HighWire Press | 9 | 1 |
| National Academies Press | 1 | 1 |
| Physician Desk Reference | 0 | - |
| UptoDate | 13 | 1 |
| **TOTAL HITS** | **115** | **17** |

**News Sources**

| **Website/Database** | **Hits** | **Unique Hits** |
| --- | --- | --- |
| Factiva | 1 | 1 |
| Google News | 2 | 2 |
| Lexis Nexis | 6 | 6 |
| ProQuest: Canadian Newstand Major Dailies | 0 | - |
| **TOTAL HITS** | **9** | **9** |

**Professional Association Websites**

| **Website** | **Hits** | **Unique Hits** |
| --- | --- | --- |
| American College of Obstetricians and Gynecologists | 0 | - |
| American Medical Association | 0 | - |
| Institute of Medicine (IOM) | 1 | - |
| National Guideline Clearinghouse (UK) | 0 | - |
| World Health Organization (WHO) | 6 | 0 |
| US Centers for Disease Control & Prevention | 9 | 0 |
| Registered Nurses’ Association of Ontario (RNAO) | 17 | 0 |
| Canadian Nurses’ Association | 14 | 0 |
| Society of Obstetricians and Gynecologists of Canada | 10 | 0 |
| Canadian Association of Midwifery | 0 | - |
| College of Family Physicians | 5 | 0 |
| Assoc. of Reproductive Health Professionals | 0 | - |
| Canadian Collaboration for Immigrant and Refugee Health (CCIRH) | 1 | 1 |
| Ontario Public Health Association (OPHA) | 5 | 1 |
| Public Health Agency Canada (PHAC) | 1 | 1 |
| **TOTAL HITS** | **69** | **3** |
| **Other (personal communication)** | **3** | **3** |
| **GRAND TOTAL** | **382** | **112** |

**3: Source Citations (N = 112)**

Alexandercikova Z, Walton MA, Chermack S, Cunningham RM, Barry KL, Blow FC: **Correlates of partner and non-partner aggression among patients with substance use disorders in an urban ED.** *J Subst Abuse* 2012, 1-13.

Arai L: **Domestic abuse and safeguarding children.** In *Working with Children and Young People: Co-constructing Practice*. Edited by O’Dell L, Leverett S. New York, Palgrave MacMillan 2011, 106-117.

Bair-Merritt MH: **Intimate partner violence.** *Pediatr Rev* 2010, **31**(4):145-150.

Barata PC: **The role of predictive models in identifying intimate partner violence in healthcare settings: a commentary.** *Am J Prev Med* 2011, **41**(2):236-237.

Beydoun HA, Beydoun MA, Kaufman JS, Lo B, Zonderman AB: **Intimate partner violence against adult women and its association with major depressive disorder, depressive symptoms and postpartum depression: A systematic review and meta-analysis.** *Soc Sci and Med* 2012, **75**(1):959-975.

Bhandari M, Sprague S, Dosanjh S, Petrisor B, Resendes S, Madden K, *et al*: **The prevalence of intimate partner violence across orthopaedic fracture clinics in Ontario.** *J Bone Joint Surg Am* 2011, **93**(2):132-41.

Bumiller K: **The nexus of domestic violence reform and social science: From instrument of social change to institutionalized surveillance.** *Annu Rev Law Soc Sci* 2010, **6**:173-93.

Buzawa ES, Buzawa CG, Stark E: **Domestic violence, health, and the health system response.** In *Responding to Domestic Violence: The Integration of Criminal Justice and Human Services*. 4th edition. Thousand Oaks: Sage; 2012:371-396.

Choo EK, Nicolaidis C, Jenkinson RH, Cox JM, McConnell KJ: **Failure of intimate partner violence screening among patients with substance use disorders.** *Acad Emerg Med* 2010, **17**(8):886-9.

Choo EK, Nicolaidis C, Newgard CD, Hall MK, Low RA, McConnell KJ, *et al*: **Association between emergency department resources and diagnosis of intimate partner violence.** *Eur J Emerg Med* 2012, **19**(2):83-88.

Chu SY, Goodwin MM, D’angelo DV: **Physical violence against US women around the time of pregnancy 2004-2007.** *Am J Prev Med* 2010, **38**(3):317-22.

Coker AL, Garcia LS, Williams CM, Crawford TN, Clear ER, McFarlane J, *et al*: **Universal Psychosocial Screening and Adverse Pregnancy Outcomes in an Academic Obstetric Clinic.** Am J *Obstet Gynec* 2012, **119**(6):1180-1189.

Coker AL, Smith PH, Whitaker DJ, Le B, Crawford TN, Flerx VC: **Effect of an in-clinic IPV advocate intervention to increase help seeking, reduce violence, and improve well-being.** *Violence Against Women* 2012, **18**(1):118-131.

Colarossi L, Breitbart V, Betancourt G: **Barriers to screening for intimate partner violence: a mixed-methods study of providers in family planning clinics.** *Perspect Sex Reprod Health* 2010, **42**(4):236-243.

Colarossi LG, Breitbart V, Betancourt GS: **Screening for intimate partner violence in reproductive health centers: an evaluation study.** *Women Health* 2010, **50**(4):313-26.

Cole TB, Flanagin A: **Theme Issue on Violence and Human Rights—Call for Papers.** *JAMA* 2012, **300**(21):2384.

Cole TB, Flanagin, A: **Evolving research on the treatment of health effects of violence and human rights abuses.** *JAMA* 2011, **308**(7):716-717.

Comes J: **Domestic violence.** In *Rosen and Barkin's 5-Minute Emergency Medicine Consult (The 5-Minute Consult Series)*. 4th edition. Edited by Schaider JJ, Barkin RM, Hayden SR, et al. Philadelphia: Lippincott, Williams, & Wilkins; 2010:332-333.

Cronholm PF, Fogarty CT, Ambuel B, Harrison SL: **RE: Qualitative benefits of screening for intimate partner violence.** *Am Fam Physician* 2012, **85**(10):Online.

Cronholm PF, Fogarty CT, Ambuel B, Harrison SL: **Intimate Partner Violence.** *Am Fam Physician* 2011, **83**(10):1165-1172.

D’Avolio DA: **System issues: challenges to intimate partner violence screening and intervention.** *Clin Nurs Res* 2011, **20**(1):64-80.

Davidov DM, Nadorff MR, Jack SM, Coben JH: **Nurse home visitors’ perceptions of mandatory reporting of intimate partner violence to law enforcement agencies.** *J Interpers Violence* 2012, **27**(12):2484-502.

Devine A, Spencer A, Eldridge S, Norman R, Feder G: **Cost-effectiveness of identification and referral to improve safety (IRIS), a domestic violence training and support programme for primary care: a modelling study based on a randomised controlled trial.** *British Med J* 2012, **2**(3):e001008.

Dichter ME, Cerulli C, Bossarte RM: **Intimate partner violence victimization among women veterans and associated heart health risks.** *Womens Health Issues* 2011, **21**(4):190-4.

Djikanovic B, Wong SLF, Stevanovic S, Celik H, Lagro-Janssen A: **Women’s expectations of healthcare professionals in case of intimate partner violence in Serbia.** *Women Health* 2011, **51**:693-708.

Djikanovic B, Wong SLF, Jansen HAFM, Koso S, Simic S, Otasevic S, *et al*: **Help-seeking behaviour of women who experienced intimate partner violence: a cross-sectional study conducted in Belgrade, Serbia.** *Fam Pract* 2012, **29**(2):189-195.

Edwardsen EA, Horwitz SH, Pless NA, le Roux HD, Fiscella KA: **Improving identification and management of partner violence: examining the process of academic detailing: a qualitative study.** *BMC Med Educ* 2011, **11**:36.

Faulkner RA, Lekander M: **Domestic violence.** In *5-Minute Clinical Consult 2012* 20th edition. Edited by Domino. FJ. Philadelphia: Lippincott Williams & Wilkins; 2012:408-409.

Feder G, Davies RA, Baird K, Dunne D, Eldridge S, Griffiths C, *et al*: **Identification and referral to improve safety (IRIS) of women experiencing domestic violence with a primary care training and support programme: a cluster randomised controlled trial.** *Lancet* 2011, **378**:1788-95.

Feinstein RE, Snavely A: **Crisis intervention, trauma, and intimate partner violence**. In *Textbook of family medicine*. 8th edition (online). Edited by Rakel RE, Rakel DP. Philadelphia: Saunders, Elsevier; 2011.

Finnegan KT, Rogers SC, Borrup K, Allen N, Saleheen H, Smith SR: **The feasibility of screening for violence in the pediatric emergency department.** *Connecticut Medicine* 2012, **76**(7):405-411.

Ford-Gilboe M, Varcoe C, Wuest J, Merritt-Gray M: **Intimate Partner Violence and Nursing Practice.** In *Family Violence and Nursing Practice 2^nd^ Edition.* Edited by Humphreys, J. and Campbell JC. New York: Springer Publishing Company; 2011: 115-153.

Gerlock AA, Grimesey JL, Pisciotta AK, Harel O: **Original research: documentation of screening for perpetration of intimate partner violence in male veterans with PTSD.** *Am J Nurs* 2011, **111**(11):26-32.

Gilbert R, Woodman J, Logan S: **Developing services for a public health approach to child maltreatment.** *International Journal of Children’s Rights* 2012, **20**:323-342.

Gilchrist G, Hegarty K, Chondros P, Herrman H, Gunn J: **The association between intimate partner violence, alcohol and depression in family practice.** *BMC Fam Pract* 2010, **11**:72.

Gottlieb AS, Shrager S: **Screening for family violence.** In *Family Violence: What Healthcare Providers Need to Know*. Edited by Fife RS, Schrager S. London: Jones & Bartlett Learning; 2012:143-52.

Gregory A, Ramsay J, Agnew-Davies R, Baird K, Devine A, Dunne D, *et al*: **Primary care identification and referral to improve safety of women experiencing domestic violence (IRIS): protocol for a pragmatic cluster randomised controlled trial.** *BMC Public Health* 2010, **10**:54.

Gucek NK, Svab I, Selic P: **The prevalence of domestic violence in primary care patients in Slovenia in a five-year period (2005-2009).** *CMJ* 2011, **52**(6):728-734.

Haegerich TM, Dahlberg LL: **Violence as a public health risk.** *Am J Lifestyle Med* 2011, **5**(5):392-406.

Haggerty LA, Hawkins JW, Fontenot H, Lewis-O’Connor A: **Tools for screening for interpersonal violence: state of the science.** *Violence and Victims* 2011, 26(6):725-737.

Hassan G, Thombs BD, Rousseau C, Kirmayer LJ, Feightner J, Ueffing E, Pottie K, Canadian Collaboration for Immigrant and Refugee Health: **Appendix 13: Intimate partner violence: evidence review for newly arriving immigrants and refugees.** *Guidelines for Immigrant Health* 2011.

Hegarty K, Glasziou P: **Tackling domestic violence: is increasing referral enough?** *Lancet* 2011, **378**(9805):1760-1762.

Hegarty KL, Doherty L, Astbury J: **Identifying intimate partner violence when screening for health and lifestyle issues among women attending general practice.** *Aust J Prim Health* 2012, **18**(4):327-331.

Hegarty KL, Gunn JM, O’Doherty LJ, Taft A, Chondros P, Feder G, *et al*: **Women’s evaluation of abuse and violence care in general practice: a cluster randomised controlled trial (weave).** *BMC Public Health* 2010, **10**:2.

Hellmuth JC, Gordon KC, Stuart GL, Moore TM: **Risk factors for intimate partner violence during pregnancy and postpartum.** *Womens Mental Health* 2012, **16**(1):19-27.

Hooker L, Ward B, Verrinde G: **Domestic violence screening in maternal & child Health nursing practice: a scoping review.** *Contemp Nurse* 2012, **42**(2).

Institute of Medicine (IOM): *Clinical Preventive Services for Women: Closing the Gaps*. Washington; National Academies Press; 2011.

Itzen C, Taket A, Barter-Godfrey S (Eds): **Violence and abuse through the life-course.** In *Domestic and Sexual Violence and Abuse: Tackling the Health and Mental Health Effects*. New York: Routledge; 2010:72-96.

Jaquier V, Hellmuth JC, Sullivan TP: **Posttraumatic stress and depression symptoms as correlates of deliberate self-harm among community women experiencing intimate partner violence.** *Psychiatry Res* 2012.

Jatoi A, Breitkopf CR: **Is spanish language a barrier to domestic violence assessment?** *J Womens Health* 2011, **20**(7):1111-6.

Jayatilleke A, Poudel KC, Jimba M: **Screening for intimate partner violence.** *JAMA* 2009, **302**(22):2434-2435.

Kam CW, Lau TCL, So FL: **Treatment and screening of intimate partner violence at a hospital's emergency department.** In *Preventing Family Violence: A Multidisciplinary Approach*. Edited by Chan KL, Aberdeen, HK. Hong Kong: University Press; 2012:239-276.

Kang, JA, Gottlieb AS, Raker CA, Aneja SS, Boardman LA**: Interpersonal violence screening for ambulatory gynecology patients.** *Obstet Gynecol* 2010, **115**(6):1159-1165.

Kataoka Y, Yaju Y, Eto H, Horiuchi S: **Self-administered questionnaire versus interview as a screening method for intimate partner violence in the prenatal setting in Japan: A randomised controlled trial.** *BMC Pregnancy Childbirth* 2010, **10**(84).

Kingsberg SA, Kellogg-Spadt S: **The essentials of assessing the sexual concerns of your female patients: barriers to discussing sexual health.** *Medscape Educ* [online] 2010, [http://www.medscape.org/viewarticle/710343_2].

Klevens J, Sadowski L[,](http://refworks.scholarsportal.info.proxy1.lib.uwo.ca:2048/refworks2/default.aspx?r=references|MainLayout::init) Kee R, Trick W, Garcia D: **Comparison of screening and referral strategies for exposure to partner violence.** *Womens Health Issues* 2012, **22**(1):e45-52.

Klevens J, Kee R, Trick W, Garcia D, Angulo FR, Jones R, *et al*: **Effect of Screening for Partner Violence on Women’s Quality of Life.** *JAMA* 2012, **308**(7):681-689

Koziol-McLain J, Garrett N, Fanslow J, Hassall I, Dobbs T, Henare-Toka TA, *et al*: **A randomized controlled trial of a brief emergency department intimate partner violence screening intervention.** *Ann Emerg Med* 2010, **56**(4):413-423.

Laisser RM, Nystro L, Lindmark G, Lugina HI, Emmelin M: **Screening of women for intimate partner violence: a pilot intervention at an outpatient department in Tanzania**. *CoAction* 2011, **4**:7288.

Lomas, Clare: **Domestic violence screening provides only ‘modest benefit’**. *Nursing Times.Net.*  Aug 9, 2009. http://www.nursingtimes.net/whats-new-in-nursing/acute-care/domestic-violence-screening-provides-only-modest-benefit/5004839.article

Lutgendorf MA, Thagard A, Rockswold PD, Busch JM, Magann EF: **Domestic violence screening of obstetric triage patients in a military population.** *J Perinatol* 2012, **32**(10):1-7.

Macy RJ, Johns N, Rizo CF: **Domestic violence and sexual assault service goal priorities.** *J Interpers Violence* 2011, **26**(16):3361-82.

Macy RJ, Rizo CF, John NB, Ermentrout DM: **Directors’ opinions about domestic violence and sexual assault service strategies that help survivors.** *J Interper Violence* 2012.

Mason R, Schwartz B: **Using a delphi method to develop competencies: the case of domestic violence.** *J Community Med Health Edu* 2012, **2**:124.

McNamara M, Walsh J: **Women's Health Issues.** In *Current Medical Diagnosis and Treatment.* Edited by Papadakis M, McPhee SJ, Rabow MW. McGraw Hill Companies; 2013:17.

Menchine MD, Vishwanath A, Arora S: **Prevalence, health and demographics of emergency department patients with diabetes.** *West J Emerg Med* 2010, **11**(5):419-22.

Miszkurka M, Zunzunegui MV, Goulet L: **Immigrant status, antenatal depressive symptoms, and frequency and source of violence: what's the relationship?** *Womens Mental Health* 2012, **15**:387-396.

Moon M. **Screening for Partner Violence Found Not Beneficial**. Elsevier Global Medical News. [Internet] August 4, 2009. [http://www.elseviermed.cn/news/detail/Screening_for_Partner_Violence_Found_Not_Beneficial]. Accessed November 10, 2012.

Moracco KE, Cole TB: **Preventing intimate partner violence: screening is not enough.** *JAMA* 2009, **302**(5):568-70.

Moraes T, Fonseca A, Soares J, Bagnoli V, Souza M, Arie W, *et al*: **Construction and validation of an instrument that breaks the silence: The impact and/or sexual violence on women’s health, are shown during climacterium.** *Menopause* 2011, **19**(1):16-22.

Nelson HD, Bougatsos C, Blazina I: **Screening women for intimate partner violence: a systematic review to update the 2004 U.S. Preventive Services Task Force recommendation.** *Ann Intern Med* 2012, **156**(11).

O’Campo P, Kirst M, Tsamis C, Chambers C, Ahmad F: **Implementing successful intimate partner violence screening programs in health care settings: evidence generated from a realist-informed systematic review.** *Soc Sci Med* 2011, **72**(6):855-866.

Oldham, R: **Commentary on “intimate partner violence education for medical students: toward a comprehensive curriculum revision”.** *South Med J* 2012, **105**(4):216-217.

O'Malley DM, Kelly PJ, Cheng A: **Family violence assessment practices of pediatric ED nurses and physicians.** *J Emerg Nur 2012*.

Onyike CU, Lyketsos CG: **Aggression and violence.** In *The American Psychiatric Publishing Textbook of Psychosomatic Medicine*. 2nd edition. Edited by Levenson JL. Arlington: American Psychiatric Publishing; 2011:153-74.

Palmer VJ, Yelland JS, Taft AJ: **Ethical complexities of screening for depression and intimate partner violence (IPV) in intervention studies.** *British Med J Pub Health* 2011, **11**(5):53.

Pignone M, Salazar R: **Disease Prevention and Health Promotion**. In *Current Medical Diagnosis and Treatment.* Edited by Papadakis M, McPhee SJ, Rabow MW. McGraw Hill Companies; 2013: 1712

Pittrof R, Goodburn E: **Should we change the focus of health promotion in sexual health clinics?** *Sex Health* 2010, **7**(4):407-410.

Pottie K, Greenaway C, Feightner J, Welch V, Swinkels H, Rashid M, *et al*: **Evidence-based clinical guidelines for immigrants and refugees.** *CMAJ* 2011, **183**(12):e824-925.

Public Health Agency of Canada: **Screening for intimate partner violence in health care settings.** In *Elder Abuse E-Bulletin* [Internet] 2010, [http://www.phac-aspc.gc.ca/ea-ma/EB/eb-eng.php#tphp].

Radtke RM, Ruf M, Gunter HM, Dohrmann K, Schauer M, Meyer E, *et al*: **Transgenerational impact of intimate partner violence on methylation in the promoter of the glucocorticoid receptor.** *Transl Psychiatry* 2011, **1**:e21.

Ranney ML, Madsen T, Gjelsvik A: **Predictors of being unsafe: participation in the behavioral risk factor surveillance system 2006 intimate partner violence module.** *J Interp Viol* 2012, **27**(1):84-102

**Recent study does not support screening for intimate partner violence: Health care workers require education to recognize signs of abuse.** *Canada NewsWire* [Internet] 2009, [http://cnw.ca/3Rjo]. Accessed October 25, 2011.

Registered Nurses’ Association of Ontario: *Woman Abuse: Screening, Identification, and Initial Response (guideline supplement)*. Ontario: International Affairs & Best Practice Guidelines; 2012.

Rhodes KV, Kothari CL, Dichter M, Cerulli C, Wiley J, Marcus S: **Intimate partner violence identification and response: time for a change in strategy.** *J Gen Intern Med* 2011, **26**(8):894-899.

Rhodes, K.V: **Taking a fresh look at routine screening for intimate partner violence: what can we do about what we know?** *MFMER* 2012, **87**(5):419-423.

Robinson-Whelen S, Hughes RB, Powers LE, Oschwald M, Renker P, Swank PR, *et al*: **Efficacy of a computerized abuse and safety assessment intervention for women with disabilities: a randomized controlled trial.** *Rehabil Psychol* 2010, **55**(2):97-107.

Saitz R, Naimi TS: **Adolescent alcohol use and violence are brief interventions the answer?** *JAMA* 2010, **304**(5):575-7.

Schilling S, Snyder A, Scribano PV: **Intimate partner violence— pediatric risks of “not asking–not telling”.** *Intimate Partner Violence* 2012, **13**(3):229-238.

Screening may provide only modest benefits. Case Management Advisor. 2009 Oct 1. Accessed October 25, 2011.

Screening may provide only modest benefits. Healthcare Benchmarks & Quality Improvement [Internet]. 2009 Nov 1[http://www.henryfordconnect.com/documents/Sladen%20Library/
HBQI-November2009.pdf]. Accessed October 25, 2011.

Screening may provide only modest benefits. Healthcare Occupational Health Management. [Internet]. 2009 Sept 1. Accessed October 25, 2011.

Selic P, Pesjak K, Kersnik J: **The prevalence of exposure to domestic violence and the factors associated with co-occurrence of psychological and physical violence exposure: a sample from primary care patients.** *BMC Publ Health* 2011, **11**:621.

Siemieniuk RAC, Krentz HB, Gish JA, Gill MJ: **Domestic violence screening: prevalence and outcomes in a Canadian HIV population.** *AIDS Patient Care STDS* 2010, **24**(12):763-70.

Sillman JS: **Diagnosing, screening, and counseling for domestic violence.** In *UptoDate* [Internet]. Edited by Fletcher SW, Sokol HN. London: University of Western Ontario; 2011.

Spangaro JM, Zwi AB, Man WYN: **Who tells and what happens: disclosure and health service responses to screening for intimate partner violence.** *Health Soc Care Community* 2010, **18**(6):671-80.

Spangaro JM, Zwi AB, Poulos RG, Man WYN: **Six months after routine screening for intimate partner violence: attitude change, useful and adverse effects.** *Women Health* 2010, **50**(2):125-43.

Spangaro JM, Zwi AB, Poulos RG: **“Persist. Persist.”: A qualitative study of women’s decisions to disclose and their perceptions of the impact of routine screening for intimate partner violence.** *Psychol Violence* 2011, **1**(2):150-162.

Sprague S, Madden K, Dosanjh S, Petrisor B, Schemtisch EH, Bhandari M, *et al*: **Screening for intimate partner violence in orthopedic patients: a comparison of three screening tools.** *J Interpers Violence* 2011, **27**(5):881-898.

Sprague, S., Madden, K., Simunovic, K., Godin, K., Pham, NK., Bhandari, K., Goslings, JS: **Barriers to Screening for Intimate Partner Violence.** *Women and health* 2012, **52**:587-605.

Stene LE, Dyb G, Tverdal A, Jacobsen GW, Schei B: **Intimate partner violence and prescription of potentially addictive drugs: prospective cohort study of women in the Oslo Health Study.** *Br Med J* 2012, **2**(1):1-8.

Taft AJ, Small R, Hegarty KL, Watson LF, Gold L, Lumley JA: **Mothers’ Advocates in the Community (MOSAIC)-non-professional mentor support to reduce intimate partner violence and depression in mothers: a cluster randomised trial in primary care.** *BMC Publ Health* 2011, **11**:178.

Taket A: **Responding to domestic violence in primary care.** *BMJ* 2012, **344**:e757.

Thomas KA, Sorenson SB, Joshi M: **Police-documented incidents of intimate partner violence among young women.** *J Womens Health* 2010, **19**(6):1079-87.

**Universal screening in health-care settings for intimate partner violence shows no significant benefit for women.** *Canada NewsWire* [Internet] 2009, [http://cnw.ca/IlGAg]. Accessed October 25, 2011.

**Universal screening for intimate partner violence may provide only modest benefits. [Internet]. 2009 Aug 5. Accessed October 17, 2012.**

Uretzki I: *Surveying Public Health Units to learn the process health units undertook to implement the Routine Universal Comprehensive Screening (RUCS) protocol*. Toronto: University of Toronto Practicum Placement 2009-2010; 2010.

Wegienka G, Bobbitt KR, Woodcroft KJ, Havstad S: **Regulatory T cells vary over bleeding segments in asthmatic and non-asthmatic women.** *J Reprod Immunol* 2011, **89**(2):192-8.

Weiss, N. S: **The withholding of test results as a means of assessing the effectiveness of treatment in test-positive persons.** *J Clin Epidemi* 2012.

Wetzstein, C. **Study debunks domestic-violence screening.** *The Washington Times* [Internet] Augusust 14, 2012. [http://www.washingtontimes.com/news/2012/aug/14/study-debunks-domestic-violence-screening/]. Accessed November 10, 2012

Wong TW: **Domestic violence in the emergency department: to screen or not to screen?** *Hong Kong* *J Emerg Med* 2010, **17**(2):107-8.

Yaffe MJ, Weiss D, Lithwick M: **Seniors' self-administration of the elder abuse suspicion index (EASI): a feasibility study.** *Journal of Elder Abuse & Neglect* 2012, **24**(4):277-292.
